# Barreiras percebidas por diretores de saúde para tomada de decisão baseada em evidências

**DOI:** 10.26633/RPSP.2017.147

**Published:** 2017-12-12

**Authors:** Leonardo Augusto Becker, Mathias Roberto Loch, Reis Rodrigo Siqueira

**Affiliations:** 1 Universidade Federal do Paraná (UFPR) Pós-Graduação em Educação Física Curitiba (PR) Brasil Universidade Federal do Paraná (UFPR), Pós-Graduação em Educação Física, Curitiba (PR), Brasil.; 2 Universidade Estadual de Londrina (UEL) Programa de Pós-Graduação em Saúde Coletiva Londrina (PR) Brasil Universidade Estadual de Londrina (UEL), Programa de Pós-Graduação em Saúde Coletiva, Londrina (PR), Brasil.; 3 Washington University in St. Louis, Brown School Prevention Research Center, St. Louis (MO) St. Louis (MO) Estados Unidos Washington University in St. Louis, Brown School, Prevention Research Center, St. Louis (MO), Estados Unidos.

**Keywords:** Tomada de decisões, planejamento em saúde, Sistema Ùnico de Saúde, Brasil, Decision making, health planning, Unified Health System, Brazil, Toma de decisiones, planificación en salud, Sistema Ùnico de Salud, Brasil

## Abstract

**Objetivo.:**

Identificar as barreiras para tomada de decisão baseada em evidências na prevenção de doenças crônicas não transmissíveis segundo a percepção dos administradores de saúde no estado do Paraná.

**Métodos.:**

Foram realizadas entrevistas por telefone com 20 diretores de regionais de saúde, por meio de um roteiro semiestruturado. As entrevistas foram realizadas em 2015 e tiveram duração média de 23 minutos. Após transcrição, o conteúdo das entrevistas foi analisado para identificação de categorias.

**Resultados.:**

Foram identificadas duas categorias de barreiras, organizacionais e pessoais. As barreiras organizacionais mais frequentes foram: “falta de planejamento e gestão” e “características regionais e culturais da população”. As barreiras pessoais mais frequentes foram: “falta de incentivo e dificuldades para trabalhar com evidências científicas” e “falta de capacitação e qualificação profissional”.

**Conclusão.:**

Sugere-se reforçar o apoio aos profissionais de saúde através de cursos de capacitação técnica que envolvam esforços políticos e científicos e que atendam as prioridades de saúde da comunidade.

## Abstract (en)

**Objective.:**

To identify the barriers to evidence-based decision-making regarding chronic noncommunicable diseases perceived by health administrators from the state of Paraná, Brazil.

**Methods.:**

Telephone interviews were performed with 20 health region directors using a semi-structured script. The interviews were performed in 2015 and had a mean duration of 23 minutes. After transcription of the interview, the contents were analyzed for identification of subject categories.

**Results.:**

Two categories were identified: organizational and personal barriers. The most frequent organizational barriers were “poor planning or management” and “regional and cultural characteristics of the population.” The most frequent personal barriers were “lack of incentive and difficulty to work with scientific evidence” and “lack of training and professional qualification.”

**Conclusion.:**

Support to health professionals should be enhanced through technical training courses linked to political and scientific efforts that address the health needs of the community.

## RESUMEN

**Objetivo.:**

Determinar los obstáculos para la toma de decisiones basada en la evidencia referentes a la prevención de enfermedades crónicas no transmisibles, según la percepción de los administradores de servicios de salud en el estado de Paraná.

**Métodos.:**

Se realizaron entrevistas telefónicas a 20 directores de unidades regionales de salud, por medio de una guía semiestructurada. Las entrevistas se efectuaron en el 2015 y duraron un promedio de 23 minutos. Después de la transcripción, se analizó su contenido para determinar los tipos de obstáculos existentes.

**Resultados.:**

Se detectaron dos tipos de obstáculos, a saber, organizacionales y personales. Los obstáculos organizacionales más frecuentes fueron la falta de planificación y de gestión y las características regionales y culturales de la población. Los obstáculos personales más frecuentes fueron la falta de incentivos, las dificultades para trabajar con pruebas científicas y la falta de capacitación y de idoneidad profesional.

**Conclusión.:**

Se sugiere reforzar el apoyo a los profesionales de salud por medio de cursos de capacitación técnica que abarquen esfuerzos políticos y científicos y atiendan las prioridades de salud de la comunidad.

Atualmente, cerca de 80% das mortes ocorridas no mundo são decorrentes das doenças crônicas não transmissíveis (DCNTs), que atingem principalmente países de renda baixa e média (1). A partir das metas globais estabelecidas pela Organização Mundial da Saúde (OMS),políticas públicas voltadas à prevenção e ao controle de DCNTs têm sido desenvolvidas (e em alguns casos implementadas) em diversos países, inclusive no Brasil. Um dos aspectos destacados nessas políticas é a importância do uso de evidências para a tomada de decisão (2).

A saúde pública baseada em evidências pode ser definida como o processo de integrar intervenções baseadas na ciência com as preferências da comunidade para melhorar a saúde das populações (3, p. 5). O emprego da tomada de decisão baseada em evidências é relatado em países de renda elevada e está associado, nesses países, ao porte populacional das cidades, à escolaridade dos gestores e à presença de equipes de trabalho qualificadas (4, 5). Todavia, a tomada de decisão baseada em evidências só se concretiza se houver uma escolha política nesse sentido (3).

Entretanto, conforme o relato de gestores de saúde, existem diversas barreiras para a implementação da tomada de decisão baseada em evidências no cotidiano dos serviços de saúde (3, 6–8). Entre as barreiras citadas estão dificuldade de acesso às evidências científicas, falta de tempo e de habilidade para desenvolver um programa baseado em evidências e falta de financiamento, estrutura e capacidade técnica. Também são citados determinantes políticos, tais como diferenças ideológicas ou *lobby* de grupos com interesses particulares (3, 6-8).

No Brasil, apesar de diversos esforços realizados para o enfrentamento das DCNTs, existem importantes desafios a enfrentar, entre os quais estão a redução da inatividade física, combate ao tabagismo, ao consumo excessivo de álcool e ao excesso de peso e obesidade (9). Dentre as iniciativas para o combate às DCNTs, têm recebido grande atenção as ações políticas, tais como vigilância, informação, avaliação, monitoramento, promoção da saúde e cuidado integral (2). Contudo, pouco se conhece sobre como ocorre a tomada de decisão baseada em evidências na implementação de programas de prevenção às DCNTs entre os gestores de saúde (2, 10). Nesse sentido, é importante identificar as barreiras percebidas pelos gestores na implementação desse processo. Vale mencionar que os gestores municipais têm papel central na execução das políticas e serviços de saúde no Brasil. Além disso, são atores privilegiados, uma vez que são, pelo menos em tese, bons conhecedores das realidades locais e informantes importantes sobre aspectos ligados às dificuldades e possibilidades dos serviços de saúde (11).

Sendo assim, o objetivo deste estudo foi identificar as barreiras para a tomada de decisão baseada em evidências na prevenção de DCNTs segundo a percep- ção dos diretores das administrações regionais de saúde no estado do Paraná, Brasil.

## MATERIAIS E MÉTODOS

O estado do Paraná, na região Sul do Brasil, possui 11 320 892 habitantes, ocupa uma área territorial de 199 880 km^2^ e é composto por 399 municípios, distribuídos em 22 regionais de saúde. O estado representa a quarta maior economia do Brasil e tem um dos melhores índices de desenvolvimento humano (IDH = 0,823) do país (12).

O presente estudo faz parte de um projeto maior, denominado “Práticas locais e o uso de evidências na prevenção de doenças crônicas não transmissíveis no estado do Paraná”. Neste estudo específico, foram utilizadas informações da segunda etapa da pesquisa, na qual foram considerados elegíveis os 22 representantes das regionais de saúde do Paraná no ano de 2015, segundo registros do Conselho de Secretários Municipais de Saúde do Paraná. Ao todo, 20 entrevistas (91,0%) foram realizadas, sendo registradas uma recusa e uma perda (um gestor não estava disponível no momento da entrevista). As entrevistas foram realizadas por telefone a partir de roteiro semiestruturado, após aprovação do Comitê de Ética em Pesquisa (nº 130 240) da Pontifícia Universidade Católica do Paraná.

O roteiro da entrevista foi desenvolvido por seis pesquisadores nacionais e internacionais com experiência e publicações na área de saúde pública e tomada de decisão baseada em evidências, inclusive na área de gestão. A entrevista compreendeu 23 questões, distribuídas em seis blocos: informações sociodemográficas; prioridades de saúde na regional de saúde; conhecimento de intervenções para prevenção de DCNTs baseada em evidências; uso de tomada de decisão baseada em evidências; barreiras para tomada de decisão baseada em evidências; e apoio das secretarias estadual e municipal para os programas de prevenção de DCNTs baseada em evidências.

As entrevistas foram realizadas por um pesquisador treinado entre os meses de março e abril de 2015, de acordo com a disponibilidade de cada gestor. As entrevistas foram gravadas e transcritas e tiveram duração média de 23 minutos. Os áudios foram posteriormente descartados. Antes do início da entrevista, os participantes foram informados sobre os procedimentos relativos ao sigilo e privacidade e declararam seu consentimento em participar.

Duas questões foram consideradas no presente estudo: a) principais dificuldades para o uso de tomada de decisão baseada em evidências; e b) percepção sobre haver ou não necessidade de capacitação dos profissionais da secretaria dos municípios para o melhor uso da tomada de decisão baseada em evidências. Caso os gestores percebessem haver necessidade de capacitação na pergunta “b”, solicitava-se que relatassem quais profissionais deveriam, na opinião deles, passar por essa capacitação.

Após o término, as entrevistas foram transcritas por dois pesquisadores auxiliares e, em seguida, o coordenador do projeto fez a conferência do áudio com a transcrição. Para propósito deste estudo, os dados que poderiam identificar os gestores foram substituídos por códigos (S1, S2, etc.), sendo os mesmos desvinculados da identificação da regional de saúde correspondente, de modo a melhor preservar a identidade dos entrevistados.

A análise dos relatos foi realizada inicialmente pelo método de análise de conteúdo (13), a partir de leituras exaustivas das questões, por dois pesquisadores de forma independente. Os relatos da pergunta “a” foram classificados em duas categorias; barreiras pessoais e barreiras organizacionais para a tomada de decisão baseada em evidências (3, 6). Os relatos que se referiam a falta de financiamento, instabilidade política, falta de planejamento e gestão, falta de estrutura, falta de recursos humanos, características das regionais e culturais da população foram inseridos como barreiras organizacionais. Por sua vez, os relatos que se referiam a dificuldades de acesso a informação, falta de incentivo, dificuldades para trabalhar com evidências científicas, falta de tempo, conflito de interesse, capacitação e qualificação profissional foram classificados como barreiras pessoais.

## RESULTADOS

Dos 20 participantes, 14 (70,0%) eram mulheres. A idade dos entrevistados variou de 27 a 57 anos e o tempo de atuação como secretário municipal de saúde variou de 12 meses a 204 meses. Quanto à formação profissional, os entrevistados distribuíam-se da seguinte forma: três enfermeiros, três licenciados (pedagogia, história e psicologia), três administradores, dois assistentes sociais, dois farmacêuticos, uma pessoa formada em direito, uma em nutrição, uma em contabilidade, um analista de sistemas, um técnico em enfermagem e dois técnicos em gestão pública. Dos 20 entrevistados, 19 (95,0%) relataram ao menos uma barreira (organizacional ou pessoal) para o processo de tomada de decisão baseada em evidências na prevenção de DCNTs.

### Barreiras organizacionais para a tomada de decisão baseada e evidências

**FIGURA 1.**
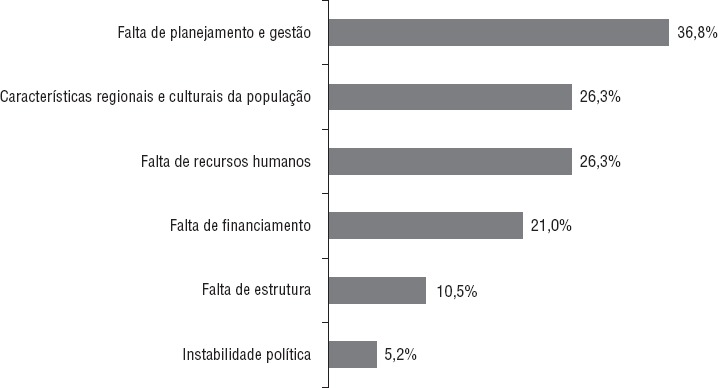
Barreiras organizacionais para o uso de tomada de decisão baseada em evidências na prevenção de doenças crônicas não transmissíveis entre os gestores de saúde do Estado do Paraná (n=19; 2015).

Como mostra a figura 1, a principal barreira organizacional citada foi a falta de planejamento e gestão, mencionada por sete (36,8%) gestores, como exemplificam as transcrições a seguir:

*A questão realmente de organização e planejamento, né! As questões de território, atenção a áreas distantes, áreas rurais, áreas distantes da unidade de saúde. Assim, poucos podem participar dessas ações né!* (S6)

*O número de atividades de programas que são colocados nas equipes acaba dificultando para os profissionais realizarem um planejamento, identificar o problema e atuar em cima do problema identificado*. (S8)

**FIGURA 2.**
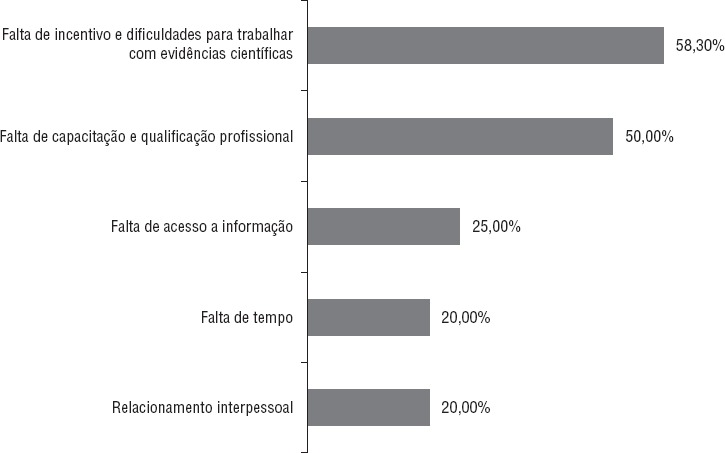
Barreiras pessoais para o uso de tomada de decisão baseada em evidências na prevenção de doenças crônicas não transmissíveis entre os gestores de saúde do Estado do Paraná (n=12; 2015).

Exemplos de outras barreiras organizacionais mencionadas aparecem a seguir:

*Aceitação da população que você vai ter que trabalhar e reeducar. Isso é o maior desafio nosso. Vou dar um exemplo: a questão do hipertenso. Nós temos na estrutura da nossa região colonização muito de descendente de europeu alemão, italiano, e eles têm hábitos de alimentação de cultura, que dificultam o nosso trabalho*. (S7)

*Hoje nós temos uma barreira grande que é a barreira financeira. Hoje o país está numa crise, por consequência os municípios estão em crise*. (S10)

*É o acúmulo de trabalho mesmo. Tem que socorrer aqui, às vezes lá. É importante ter o profissional na medida certa*. (S13)

*Acho que falta um pouco de estrutura. Faltam algumas ferramentas que são indispensáveis para desenvolver aquela ação ou aquele trabalho. O serviço público de saúde não tem toda essa estrutura*. (S11)

*Há muita rotatividade de gestor né! Esses gestores, como eu disse pra você, a grande maioria são nomeados por questões políticas e não por qualificação profissional*. (S12)

### Barreiras pessoais para a tomada de decisão baseada e evidências

Barreiras pessoais foram mencionadas por 12 (60,0%) gestores. A barreira pessoal mais frequente foi a falta de incentivo e as dificuldades para trabalhar com evidências científicas, relatada por sete (58,3%) gestores (figura 2):

*Falta incentivo, na verdade informações mais transparentes, onde no meu município eu possa encontrar essas informações! Onde estão esses dados baseados em evidências? como que eu faço? de que jeito é?* (S20)

Outras barreiras pessoais, como falta de capacitação e acesso à informação, são citadas nas transcrições a seguir:

*O desconhecimento multiprofissional de como se faz. Os profissionais não estão capacitados para fazer uso dessas ferramentas. Tanto o estado quanto união e o município da gestão não incentiva ou não conhece como faz isso*. (S20)

*Falta do conhecimento e divulgação. Uma maior capacitação para municípios, pois é quem está na ponta né! Se discute muito dentro do estado e do Ministério os programas, mas o que interessa é o que chega no município*. (S11)

*Acho que é o próprio fornecimento da informação. O conhecimento mais aprofundado desta nova metodologia. Eu acho que é o desconhecimento, aliás, o pouco conhecimento, a falta de capacitação em relação a isto, a falta de profissionais capacitados para estar realizando este trabalho*. (S17)

*Acho que uma das barreiras seria essa de ter a informação rápida e a conteúdos claros para trabalhar com equipe multidisciplinar*. (S9)

*Então acho que uma das barreiras seria essa de trabalhar com equipe multidisciplinar cada um dentro das suas prioridades.* (S9)

### Necessidade de capacitar os profissionais das secretarias para a tomada de decisão baseada em evidências

Todos os gestores relataram a necessidade de capacitar a equipe de trabalho. Em relação às pessoas que deveriam ser capacitadas, os secretários reportaram que a capacitação precisa ser ampla, envolvendo profissionais administrativos e profissionais que estão mais próximos da população, como equipe multiprofissional e agentes de saúde:

*Tem que ser um profissional de cada área, dentista, enfermeiro, agente comunitário, médicos, até o secretário de saúde, departamento administrativo. Porque daí trabalha integrado né! Todo mundo sabe que está fazendo né!* (S4)

*Os profissionais que estão à frente da atenção. De estruturação, seriam os enfermeiros, médicos, os agentes de saúde também, porque eles que estão no dia a dia das comunidades e os próprios profissionais da gestão.* (S6)

Outros profissionais apontaram a necessidade de capacitação dos profissionais de gestão:

*Eu acho que a equipe administrativa, a equipe pensante da secretaria de saúde seria o público alvo*. (S9)

*A parte da gestão e a parte administrativa, pois a parte técnica tem capacitação continuada, agora a parte da gestão falta um pouco*. (S19)

## DISCUSSÃO

O presente estudo incluiu 20 dos 22 gestores regionais de saúde no estado do Paraná, sendo que a maioria tinha experiência no cargo. Ficou evidente a grande diversidade na formação profissional desses gestores, o que de algum modo é esperado, uma vez que o setor saúde abrange muitas áreas profissionais. Além disso, por seu caráter intersetorial (14, 15), a área da saúde tem espaço para que pessoas sem formação específica tenham um papel importante na definição das ações de saúde, inclusive exercendo cargos de gestão (14, 15). Ainda que a formação específica seja desejável, deve-se considerar que a disponibilidade de cursos e pessoal capacitado pode ser um desafio, especialmente em municípios de pequeno porte. Nesse sentido, iniciativas têm sido implementadas para ampliar e democratizar o acesso à formação em saúde pública (16), o que pode ajudar a modificar este cenário.

Por outro lado, é importante mencionar que, em países desenvolvidos, o emprego da tomada de decisão baseada em evidências tem sido descrito entre gestores que possuem maior experiência em saúde pública e elevada escolaridade, além de estar associado ao porte populacional das cidades (5, 17). Portanto, a diversidade e a baixa especificidade de formação em saúde pública na presente amostra podem explicar, ao menos em parte, a alta frequência de barreiras organizacionais percebidas pelos gestores. Em sua maioria, os gestores relataram a necessidade de maior planejamento e gestão, corroborando a necessidade de formação na área de saúde pública. Ademais, vale mencionar que a formação em saúde pública pode não estar presente, ou pode ser pouco aprofundada, nos cursos de graduação em saúde, dado que esses têm foco quase que exclusivo na atuação técnica mais específica das respectivas profissões (15).

Nesse sentido, ressalta-se que os secretários municipais de saúde têm um papel central na execução das políticas de saúde, já que são responsáveis por organizar e assegurar o direito a saúde – dado que a municipalização é uma das diretrizes estratégicas do Sistema Único de Saúde (SUS) (18). Entretanto, a nomeação de cargos públicos somente pelo critério político, sem considerar a questão técnica (11), pode gerar dificuldades para o planejamento e a gestão. Fontes que podem ser utilizadas para ampliar recursos e condi- ções para a gestão são órgãos como Conselho Nacional de Secretários de Saúde (CONASS), Conselho Nacional de Secretarias Municipais de Saúde (CONASEMS) e Conselho de Secretarias Municipais de Saúde (COSEMS) dos estados, que possuem equipe técnica e buscam enfoque em uma gestão participativa, que articule questões políticas, planejamento de programas e promoção da saúde (19).

A insuficiência de financiamento também foi uma barreira reportada pelos gestores para a tomada de decisão baseada em evidências. De fato, os investimentos cresceram consideravelmente nos últimos anos, especialmente no caso dos tratamentos médicos, muitas vezes em função da ênfase na atenção secundária e terciária (20, 21). Como consequência, são escassos os recursos destinados a formação e gestão nos serviços, inclusive sobre prevenção de DCNTs. Ainda que haja interesse e demanda por formação em tomada de decisão baseada em evidências relacionada às DCNTs, a complexidade do financiamento da saúde pública é um desafio para os gestores nos diferentes níveis de governo (municipal, estadual e federal). Além disso, a recente queda do produto interno bruto (PIB) nacional, com impacto direto na receita, aliada ao aumento na proporção de capital privado no setor saúde do Brasil, tem limitado o acesso universal à saúde (22). Portanto, o cenário de financiamento insuficiente e menor acesso aos serviços de saúde pode, em parte, explicar os relatos de falta de estrutura adequada, falta de recursos humanos, excesso de trabalho das equipes e a descontinuidade das ações, inclusive muitas vezes aquelas que apresentam bons resultados. De qualquer modo, há de se considerar que as demandas em saúde das populações são, em geral, maiores do que a capacidade técnica dos serviços de saúde de atendê-las. Isso muitas vezes vai para além das questões financeiras e inclui também as limitações técnicas e humanas da área. Considerando esse cenário, a implementação de tomada de decisão baseada em evidências em um contexto que considere a importância da atenção básica pode ser importante para a efetivação de um sistema mais resolutivo e humano (21, 22).

Os achados das barreiras organizacionais parecem ser reforçados pelas barreiras pessoais, das quais a “falta de incentivo e dificuldades para trabalhar com evidências científicas” foi a mais citada. Isso se reflete na dificuldade dos gestores de identificarem as melhores evidências científicas, as prioridades de saúde da população e as intervenções necessárias a serem realizada. Talvez isso ocorra devido às inúmeras atribuições do cargo, o que pode levar os gestores a priorizarem ações burocráticas (8). Por outro lado, verifica-se a dificuldade que os pesquisadores têm para melhorar a disseminação das pesquisas, traduzindo as principais informações para aqueles que implementam políticas públicas (23), havendo assim uma clara necessidade de maior aproximação entre o mundo “acadêmico” e a gestão em saúde. Para tal, poderiam ser desenvolvidas redes de contato envolvendo gestores e investigadores da área de saúde pública (24). Nesse sentido, para melhorar o acesso a evidências, é importante criar iniciativas como os observatórios de tecnologias de informação em serviços de saúde, que apoiam ações nas redes de saúde, de forma semelhante ao que ocorre em países desenvolvidos (25). Além disso, os gestores podem utilizar ferramentas disponibilizadas pelo Ministério da Saúde, como por exemplo o portal Saúde baseada em evidências (26).

Outra importante barreira reportada foi a “falta de capacitação e qualificação profissional”, reforçando a necessidade da formação na área de saúde pública, corroborando os achados da literatura, onde os gestores identificam dificuldades de acesso e interpretação de estudos científicos (24). Vale mencionar que, no Brasil, foi criada a Política Nacional de Reorientação da Formação do Profissional de Saúde, que busca aproximar a saúde da educação continuada e reorientar as universidades e os serviços de saúde para formação dos profissionais de saúde (27). Como reportado nesse estudo, existe a necessidade de uma melhor qualificação dos profissionais de saúde, tanto aqueles que estão mais próximos da população (por exemplo, médicos, enfermeiros, agentes comunitários e profissionais do núcleo de apoio a saúde da família), quanto dos profissionais que estão na gestão. Fica clara a importância da educação permanente dos profissionais de saúde para o desenvolvimento e planejamento das ações em saúde, inclusive porque existem evidências de que a educação permanente melhora a avaliação dos indicadores de saúde, incentiva o desenvolvimento de uma gestão mais compartilhada e melhora o acesso à informação (11, 28). Além disso, para minimizar as barreiras, poderiam ser propostas parcerias com pesquisadores e com outros gestores, objetivando uma capacitação técnica de qualidade (8). É preciso considerar que as barreiras pessoais podem até ser menos frequentes do que as barreiras organizacionais, mas, talvez, seja mais difícil para os participantes dimensionarem adequadamente as barreiras pessoais. De qualquer forma, as barreiras pessoais precisam ser consideradas na proposição de eventuais capacitações.

A educação permanente e de qualidade requer condições de trabalho e disponibilidade de tempo da equipe. Todavia, os gestores reportaram a “falta de tempo”. Certamente, isso afeta a qualidade dos serviços prestados no SUS. Para que a tomada de decisão baseada em evidências seja efetivada, são necessárias mudanças na dinâmica de trabalho: é preciso reservar tempo para pesquisar, estudar e planejar questões específicas para o processo de tomada de decisão. Nesse sentido, é preciso reorientar os profissionais de saúde e os gestores para o planejamento e organização do fluxo de atendimento local (29). Novamente, observa-se que a falta de tempo é associada a falta de planejamento e de gestão.

As barreiras organizacionais e pessoais parecem reforçar a necessidade de capacitação profissional da equipe de trabalho de modo geral, reportada pela totalidade dos entrevistados, o que é coerente com os princípios e diretrizes do SUS que enfatizam a educação permanente (27). Porém, esse processo deve se dar na perspectiva da função de apoio com características específicas para cada localidade (29). Contudo, observa-se que, mesmo quando os profissionais são capacitados, nem sempre conseguem implementar ações baseadas em evidências nos locais de serviços. Nesse sentido, propor capacitação profissional específica, adequadas às realidades das equipes e profissionais, pode ter efeitos positivos (30).

Os resultados deste estudo fornecem importantes informações sobre as barreiras percebidas para o emprego de tomada de decisão baseada em evidências na prevenção de DCNTs entre secretários municipais de saúde do estado do Paraná. No momento da entrevista, os entrevistados exerciam um cargo de grande importância (diretores das respectivas regionais de saúde), sendo certamente observadores privilegiados e estratégicos a respeito das especificidades da organização do sistema de saúde de suas respectivas regiões. Contudo, cabe notar que os resultados derivam somente dos relatos dos gestores; não foram empregadas outras estratégias de busca de informação, como observações e entrevistas com trabalhadores dos serviços de saúde, usuários e mesmo análise documental, por exemplo, das atas das reuniões das regionais de saúde. Além disso, a falta de estudos realizados no Brasil sobre o tema dificultou a melhor compreensão dos achados. Também é importante destacar a escolha por apresentar uma visão mais “genérica” e “panorâmica” dos resultados, inclusive em função do estado da arte na literatura latino-americana. Estudos futuros poderiam se apropriar de referenciais mais específicos e buscar um olhar mais aprofundando sobre algum ponto da temática.

Em conclusão, as principais barreiras organizacionais reportadas foram “a falta de planejamento e gestão” e“características regionais e culturais da população”. Das barreiras pessoais, as mais citadas foram “a falta de incentivo e dificuldades para trabalhar com evidências científicas” e “a falta de capacitação e qualificação profissional”. Com base nos resultados deste estudo, sugere-se reforçar o apoio aos profissionais de saúde através de cursos de capacita- ção técnica a partir do conceito de saúde pública e tomada de decisão baseada em evidências para prevenção DCNTs. Parece claro que tal reforço depende fundamentalmente da questão política, e, para tal, é necessário que não apenas os gestores municipais reconheçam a importância da tomada de decisão baseada em evidências no contexto da prevenção das DCNTs – mas que também outros atores, desde pessoas que participam das atividades ligadas ao controle e participação social, até os gestores ligados às secretarias municipais e Ministério da Saúde, compartilhem essa convicção. Por fim, recomenda-se a realização de outros estudos sobre o processo de tomada de decisão baseada em evidências que enfoquem aspectos que dificultam ou favorecem esse processo e que busquem verificar o impacto dessa estratégia nos indicadores de saúde da população.

### Agradecimentos

Agradecemos aos membros do Grupo de Pesquisa em Atividade Física e Qualidade de Vida (GPAQ) por terem auxiliado na coleta de dados; à Fundação Araucária do Estado do Paraná e ao Conselho Nacional de Desenvolvimento Científico e Tecnológico (CNPQ) pelo financiamento da pesquisa; e ao Conselho Estadual de Secretários Municipais de Saúde do Paraná (COSEMS-PR) por terem auxiliado na etapa de planejamento do estudo.

### Declaração de responsabilidade

As opiniões expressas no manuscrito são de responsabilidade exclusiva dos autores e não refletem necessariamente a opinião ou política da RPSP/PAJPH ou da Organização Pan-americana de Saúde (OPAS).
